# Gender disparities in pediatric research: a descriptive bibliometric study on scientific authorships

**DOI:** 10.1038/s41390-022-02010-1

**Published:** 2022-03-28

**Authors:** Katja Böhme, Doris Klingelhöfer, David A. Groneberg, Michael H. K. Bendels

**Affiliations:** Division of Computational Medicine, Institute of Occupational Medicine, Social Medicine and Environmental Medicine, Goethe-University, Frankfurt, Germany

## Abstract

**Background:**

The proportion of women in medicine, especially in pediatrics, is noticeably increasing. Yet, leadership positions are predominantly occupied by men.

**Methods:**

Academic authorships of 156,642 pediatric original research articles were analyzed with regard to gender disparities. The evaluation included the proportion of female authorships (FAP), distributions over first-, co- and last-authorships, gender-related citation rates, a productivity analysis and investigations on journals, countries and pediatric sub-disciplines.

**Results:**

In all, 46.6% of all authorships in pediatric research were held by female authors. Women held relatively more first-authorships (FAP = 52%) and had higher odds for first- (OR = 1.3) and co- (OR = 1.11) authorships, compared to men. The Prestige Index of −0.13 indicated an underrepresentation of female authors at prestigious first- and last-authorships. Citation rates were not affected by the gender of the key authors. At the country-level pronounced gender-related differences were detected. The time trend showed increasing female prospects forecasting a female-dominated Prestige Index of 0.05 in 2023.

**Conclusion:**

The integration of women in pediatric research has advanced. Opportunities for female authors differ at the country-level, but overall women are lacking in leadership positions. Improving career opportunities for women in pediatric research can be expected in the coming years.

**Impact:**

- There is a measurable progress in the integration of female scientists.
- Gender-neutrality is partially achieved in pediatric research with yet a female underrepresentation in leading positions.
- Our descriptive study presents gender-related dynamics in pediatric research that forecast improving career opportunities for female scientists.

## Introduction

Pediatrics is dominated by women.^1^ The feminization of medicine is widely apparent, but particularly noticeable in pediatrics. Historically, the sociological assignment of childcare to the role of women made it easier for female doctors to enter pediatrics.^2^ Over the past decades, the proportion of women in pediatrics has steadily increased.^2^ Today, >70% of the pediatric residents in the US are female.^1^ However, gender inequity is evident when considering leadership positions, such as pediatric department chairs, with a female proportion of 26.3% in 2020.^3^

In this study, we examine the integration of female scientists in pediatric research based on scientific authorships. We anticipate that early-career researchers primarily publish as first- or co-authors in original articles, while senior researchers preferably publish as last-authors.^4–6^ First- and last-authorships are associated with a certain prestige and are considered a type of currency in academic medicine.^4,7^

Gender disparities have recently drawn a lot of interest and were evaluated for several medical subjects.^5,8–20^ Overall, female authors are numerically under-represented in academic medicine and reach lower citation rates than their male colleagues.^12,21,22^ Previous research on selected pediatric journals has shown an increasing proportion of female authors.^5,20^ Fishman et al.^5^ examined three pediatric high-impact journals. They detected an overrepresentation of women at first-authorships with 57.7% and an underrepresentation of women at last-authorships with 38.1% in 2016, in the selected journals.^5^ Regarding perspective-type articles in four pediatric high-impact journals, Silver et al.^23^ documented a female underrepresentation at first-, co- and last-authorships.^23^ The analysis of three Latin American pediatric Journals by Otero et al.^20^ on the other hand revealed relatively high proportions of female authors.^20^ In their data set 59.9% of all authors, 54.4% of first-authors, and 48% of last-authors were women in 2015.^20^

To obtain representative results for the entire field of pediatric science, we analyzed original research data from a total of 400 journals with >690,000 authorships. We evaluated the temporal development and gender-specific citation numbers, and compared gender distributions of countries, journals and pediatric sub-disciplines. Finally, we provide a forecast for the near future.

## Materials and methods

### Data acquisition and integration

Pediatric English-language original research articles published between January 1, 2008 and December 31, 2018 form the basis of this study. The data were acquired from the category ‘Pediatrics’ of the Web of Science Core Collection. The integration and bibliometric analysis was performed by Gendermetrics.Net,^24^ a SQL server-based software.^24^ The process included the unification of authors by grouping them by their first and last name. In total, 156,642 articles published in 400 journals written by 363,518 authors from 182 countries were acquired (bibliometric overview in Supp. Fig. 1).^24^

### Gender determination

The gender determination was algorithmically conducted through Gendermetrics.Net by evaluation of the authors’ first name(s).^24^ We found 146,453 (=40.3%) female authors and 129,729 (=35.7%) male authors. 16,673 (=4.6%) authors had unisex first names and 70,663 (=19.4%) first names could not be identified. Authors with unisex or non-identified forenames and their corresponding authorships (in total 162,400 authorships) were excluded from the gender analysis. The remaining 690,436 male and female authorships formed the data basis of the gender analysis.

For sub-analyzes, data were grouped by different criteria (publication year, country of authorship, journal, number of authors per article, subject areas). In order to ensure the statistical validity, only groups with at least 750 male/female authorships and a gender detection output of a least 60% male and female authorships were included.^13^ The application of the stated criteria led to an exclusion of 287 journals from the journal-specific analysis because of too low numbers of detected female/male authorships. From the country-specific analysis China and South Korea were excluded owing to too high rates of unisex names.

Furthermore, subject areas were defined by tags of Web of Science and formed the basis for the corresponding sub-analysis.

### Proportion of female authorships and female authorship odds ratio

The subjects of the analysis were first-, co- and last-authorships.^11^ Single authorships were rated as first-authorships, authorships of articles with two authors were counted as first- and last-authorship.^11^ Co-authorships described all authorships between one first- and one last-authorship.^11^

The female authorship proportion (FAP) is the percentage of female authorships out of all female and male authorships (FAP = Authorships_female_/Authorships_female+male_).^12^

In contrast, the female authorship odds ratio (FAOR) describes the relative distribution of female authors over first-, co- and last-authorships compared to men.^12^ In order to determine the FAOR for first-authorships for instance, the female odds for first-authorships are divided by the male odds for first-authorships (FAOR_first_ = Female Odds_first_/Male Odds_first_).^12^ FAORs for co- and last-authorships were calculated in the equivalent way. A FAOR > 1 represents higher female than male odds for the corresponding authorship.^25^ FAORs are determined with a confidence level of 95%.^13^

To provide a good overview, a FAOR triplet is used to present the relative chance distributions.^13^ A triplet of (+**, =,** –), for example, indicates *significantly* higher (+) female odds to secure first-authorships, equal (=) odds for co-authorships and *significantly* lower (-) female odds for last-authorships, compared to men.^13^

Summarized, the FAP measures the proportion of female authorships, whereas the FAOR gives information about distribution odds over first-, co- and last-authorships.^11^

### Prestige Index

The Prestige Index (PI) is a measure of the distribution of prestigious authorships between male and female authors.^13^ Bendels et al.^13^ introduced and defined the Prestige Index “as the prestige-weighted average of the FAOR excess *ε*_t_ that is calculated over all authorship types *t*[…] with the weighting factor *w*_t_”.^12^ It was computed by *ε*_t_ = *w*_t_ (FAOR_t_ – 1) if FAOR_t_ ≥ 1 and *ε*_t_ = *w*_t_ (1 – 1/FAOR_t_) if FAOR < 1.^12^ Since first- and last-authorships are associated with a high reputation they are weighted positively with *w*_first_ = *w*_last_ = 1, while co-authorships are weighted with *w*_co_ = −1.^6,13^ Thereby the Prestige Index increases with a higher female odds ratio (OR) for first- or last-authorships and with a lower female OR for co-authorships.^13^ A gender-neutral prestige distribution is indicated by a Prestige Index of 0, while a positive (negative) Prestige Index states that female authors hold relatively more (less) prestigious authorships than men.^13^

### Analysis of data

Average annual growth rates (AAGR) were determined by computing the mean values of *n* annual growth rates.^10^ The calculations also served the temporal linear predictions of the article count, the FAP, the FAOR and PI.^12^

In the respective sub-analyzes (countries, journals, subject areas) we computed linear correlations of parameters by applying the Pearson correlation.^13^ We excluded 10 of 113 considered journals from the journal-specific sub-analysis due to a missing 5-year-impact-factor. Moreover, we applied a Kruskal–Wallis and a post hoc multi-comparison test to test the null hypothesis, whether the not normally distributed citation rates were drawn from the same distribution.^12^ Significance thresholds were set at 0.05.^12^

## Results

### Status quo and temporal development

**Fig. 1:**
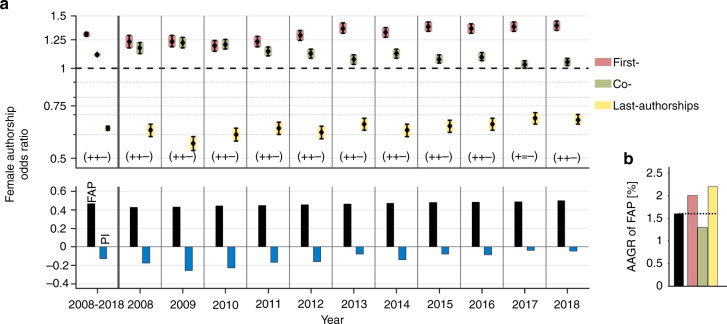
Time trend of female authorships on the global level. **a** The female authorship odds ratio (FAOR, top) with associated FAOR triplets, the proportion of female authorships (FAP, bottom) and the Prestige Index (PI, bottom) are depicted averaged over time and by year from 2008 to 2018. The average FAP is 46.6% and has been increasing over time from 42.5% in 2008 up to 49.9% in 2018. The negative PI (minimum in 2009) approaches a gender-neutral distribution of renowned authorships. Owing to increasing female odds for first- and last-authorships and decreasing female odds for co-authorships the PI rises up to a maximum of −0.05 in 2018. The FAOR-pattern is almost exclusively characterized by the triplet (+, +, –), indicating significantly higher odds ratios (+) for female first- and co-authorships and significantly lower odds ratios (–) for female last-authorships. **b** The average annual growth rate (AAGR) of the FAP exhibits a yearly increase of 1.6% on average with highest growth rates for last- and first-authorships, which are associated with a higher prestige.

Female authors are under-represented in pediatric research with a FAP of 46.6% at the global level (Fig. 1a). Female authors hold 52.0% first-, 47.6% co- and 37.5% last-authorships. FAORs are 1.30 for first-authorships (CI = 1.28–1.32), 1.11 for co-authorships (CI = 1.1–1.12) and 0.63 for last-authorships (CI = 0.62–0.64). The corresponding FAOR-pattern is accordingly characterized by the triplet (+, +, –). Proportionally, women secure less prestigious authorships than men as indicated by a global Prestige Index of −0.13.

The FAP steadily increased over the last decade from 42.5% in 2008 to 49.9% in 2018 with an AAGR of 1.6%. The highest growth rates are found for last- and first-authorships with 2.2% and 2.0%, respectively (Fig. 1b). The AAGR of female co-authorships is 1.3%.

Female odds to hold first- and last-authorships have increased, while female odds for co-authorships have decreased since 2008. As a result of this drift, the Prestige Index has risen from its minimum of −0.26 in 2009 and has almost approached gender-neutrality at −0.05 in 2018.

### Differences across countries

At the country-level, we find a FAP ranging from 21.8% in Japan, 22.7% in Saudi Arabia and 33.3% in Pakistan to 62.8% in Poland, 63.0% in Serbia, and 65.9% in Portugal (Table 1). The Prestige Index varies between a minimum of −0.90 in Italy, −0.80 in Colombia, and −0.77 in Japan, to higher indices of 0.39 in Sweden, 0.42 in Denmark, and then climaxes at a maximum of 0.54 in the Netherlands. Regarding the distribution of authorships, most countries show higher or equal odds ratios for women to be first- or co-authors while men have higher odds to be last-authors. Five countries (Singapore, Kenya, Portugal, Croatia and Tunisia) are characterized by gender-neutrality regarding authorship odds (FAOR triplet (=, =, =)). Remarkably, Ireland is the only country characterized by higher female odds to secure last-authorships compared to men.

**Table 1:** Classification by country.

Country name	Prestige Index	Proportion of female authorships	FAOR triplet	No. articles	No. authorships
Netherlands	0.54	48.7%	(+, –, –)	5266	18,814
Denmark	0.42	46.1%	(+, –, –)	1555	4893
Sweden	0.39	54.0%	(+, –, –)	3263	8958
Norway	0.28	51.9%	(+, –, –)	1434	4301
Ireland	0.16	50.8%	(=, =, +)	1133	3052
Australia	0.03	52.9%	(+, =, –)	6819	22,353
Singapore	0.03	48.9%	(=, =, =)	485	1000
Iran	–0.01	38.7%	(+, =, –)	2751	10,531
Finland	–0.03	58.7%	(+, =, –)	1651	6528
India	–0.04	37.2%	(+, =, –)	6667	18,165
Brazil	–0.05	59.1%	(+, =, –)	3212	12,986
Kenya	–0.05	44.1%	(=, =, =)	327	864
Switzerland	–0.07	41.8%	(+, =, –)	2327	6925
Portugal	–0.07	65.9%	(=, =, =)	776	2731
Canada	–0.11	50.7%	(+, +, –)	9373	30,547
South Africa	–0.11	46.4%	(=, =, –)	1240	2632
New Zealand	–0.11	48.0%	(+, =, –)	1083	2934
Croatia	–0.11	57.2%	(=, =, =)	305	1072
United States	–0.13	47.9%	(+, +, –)	64186	260,726
United Kingdom	–0.13	45.7%	(+, +, –)	11851	30,885
Germany	–0.13	36.3%	(+, =, –)	6265	22,293
Tunisia	–0.14	56.7%	(=, =, =)	199	831
Turkey	–0.15	46.3%	(+, +, –)	7473	34,112
Austria	–0.16	42.7%	(+, =, –)	1265	3765
Chile	–0.18	55.8%	(=, =, –)	550	1738
Belgium	–0.26	50.1%	(+, +, –)	1883	4955
Pakistan	–0.29	33.3%	(=, =, –)	310	823
Egypt	–0.30	40.6%	(–, +, =)	1296	3587
Serbia	–0.31	63.0%	(=, +, –)	297	1207
France	–0.32	49.8%	(+, +, –)	4088	17,225
Mexico	–0.33	47.4%	(=, +, –)	683	2510
Israel	–0.37	43.7%	(=, +, –)	2685	10,009
Poland	–0.37	62.8%	(=, +, –)	1291	4924
Argentina	–0.40	59.5%	(=, +, –)	786	3487
Czech Republic	–0.41	47.1%	(=, +, –)	445	1271
Spain	–0.45	55.6%	(=, +, –)	3139	12,917
Hungary	–0.45	44.8%	(+, =, –)	409	1319
Saudi Arabia	–0.52	22.7%	(=, +, –)	832	1939
Greece	–0.54	52.4%	(=, +, –)	1150	4615
Japan	–0.77	21.8%	(+, +, –)	6012	34,293
Colombia	–0.80	46.6%	(=, +, –)	287	777
Italy	–0.90	55.2%	(=, +, –)	6748	33,198

The countries are arranged in descending order to their Prestige Index.

A country’s FAP and its Prestige Index are not linearly correlating (*r* = 0.18, *P* > 0.05).

### Differences across journals

The FAP range on the journal-level starts at 19.4% in *Journal of Pediatric Orthopedic-Part B*, 24.5% in *Journal of Neurosurgery—Pediatrics*, and 25.3% in *Journal of Pediatric Orthopedics* and ascends up to 83.2% in *Journal of Pediatric Nursing—Nursing Care of Children & Families*, 84.2% in *Journal of Perinatal & Neonatal Nursing*, to a maximum of 84.8% in *Journal of Pediatric Health Care* (Table 2).

**Table 2:** Classification by journals.

Journal name	Prestige Index	Proportion of female authorships	FAOR triplet	No. of articles	No. of authorships
Journal of Perinatal & Neonatal Nursing	0.64	84.2%	(+, =, –)	373	894
Journal of Pediatric Nursing—Nursing Care of Children & Families	0.57	82.3%	(+, –, =)	830	2823
Journal of Pediatric Health Care	0.54	84.8%	(+, =, –)	491	1614
Maternal and Child Nutrition	0.5	65.8%	(+, –, –)	784	3685
Physical & Occupational Therapy in Pediatrics	0.44	81.6%	(+, –, =)	275	1043
Birth-Issues in Perinatal Care	0.29	72.8%	(+, –, =)	444	1732
Journal of Pediatric and Adolescent Gynecology	0.28	69.7%	(+, –, =)	797	2869
Journal of Child Health Care	0.25	71.6%	(+, =, =)	400	1326
International Journal of Pediatrics-Mashhad	0.23	39.6%	(+, –, =)	699	2711
Pediatrics and Neonatology	0.21	42.8%	(+, –, =)	642	2142
Pediatric and Perinatal Epidemiology	0.20	56.9%	(+, –, –)	678	3618
International Journal of Pediatric Dentistry	0.19	54.9%	(+, =, –)	657	2541
Developmental Medicine and Child Neurology	0.18	57.2%	(+, –, –)	1383	6973
European Child & Adolescent Psychiatry	0.18	54.9%	(+, –, –)	929	5006
Journal for Specialists in Pediatric Nursing	0.16	82.4%	(+, =, –)	289	834
Journal of Adolescent Health	0.12	61.9%	(+, –, –)	2132	9897
Pediatric Annals	0.10	55.6%	(+, =, –)	782	1643
Journal of the Pediatric Infectious Diseases Society	0.10	51.6%	(+, =, =)	299	1950
International Journal of Pediatric Obesity	0.09	54.3%	(+, =, –)	318	1326
Pediatric Diabetes	0.07	53.7%	(+, =, –)	1104	6256
Pediatric Physical Therapy	0.06	72.2%	(+, =, –)	452	1617
Pediatric Allergy and Immunology	0.05	46.9%	(+, =, –)	1008	5913
Journal of Tropical Pediatrics	0.05	43.7%	(+, =, –)	831	3269
Pediatric and Developmental Pathology	0.05	49.6%	(+, =, –)	706	2901
Pediatric Rheumatology	0.05	55.4%	(+, =, –)	430	2590
Breastfeeding Medicine	0.04	67.7%	(+, =, –)	768	2484
Jornal de Pediatria	0.04	64.3%	(+, =, –)	661	3024
Pediatric Transplantation	0.03	39.1%	(+, =, –)	1715	9502
Childhood Obesity	0.03	70.4%	(+, =, –)	424	2098
Pediatric Dentistry	0.02	49.0%	(+, =, –)	667	2278
Journal of Human Lactation	0.02	75.2%	(+, =, –)	628	2347
Journal of Pediatric Surgery Case Reports	0.01	30.7%	(+, =, –)	707	2792
Case Reports in Pediatrics	0.01	47.4%	(+, =, –)	322	1227
Pediatric Research	0.00	45.5%	(+, =, –)	2180	13,318
Pediatric Dermatology	–0.01	55.5%	(+, =, –)	2257	8990
Child and Adolescent Mental Health	–0.01	63.7%	(=, =, =)	320	1218
Child and Adolescent Psychiatry and Mental Health	–0.01	54.6%	(+, =, –)	248	1122
Pediatric Blood & Cancer	–0.03	47.2%	(+, =, –)	4116	26,704
Pediatric Surgery International	–0.03	28.9%	(+, =, –)	1971	8499
Indian Pediatrics	–0.03	40.7%	(=, =, –)	1390	3635
Neuropediatrics	–0.03	49.1%	(+, =, –)	576	2092
Developmental Neurorehabilitation	–*0.04*	62.7%	(=, =, =)	500	2091
Bmc Pediatrics	–0.05	52.5%	(+, =, –)	2008	10,808
Jama Pediatrics	–0.05	48.8%	(+, =, –)	641	4887
Journal of Pediatric Hematology Oncology	–0.06	47.7%	(+, =, –)	2312	11,839
Pediatric Allergy Immunology and Pulmonology	–0.07	56.6%	(+, =, –)	272	1098
Pediatrics & Child Health	–0.08	57.1%	(+, =, –)	434	1600
Journal of Clinical Research in Pediatric Endocrinology	–0.08	53.0%	(+, =, –)	385	2024
Journal of Pediatrics and Child Health	–0.09	52.1%	(+, =, –)	1592	6507
Acta Paediatrica	–0.10	50.0%	(+, =, –)	3027	10,700
Pediatric Neurology	–0.11	45.9%	(+, =, –)	1713	8206
Journal of Developmental and Behavioral Pediatrics	–0.11	63.3%	(+, =, –)	776	3718
Archives of Disease in Childhood	–0.12	49.6%	(+, =, –)	1767	6108
Pediatric Critical Care Medicine	–0.12	40.8%	(+, =, –)	1668	9941
Cardiology in the Young	–0.13	35.7%	(+, =, –)	1935	8185
Pediatric Emergency Care	–0.14	43.6%	(+, +, –)	2078	8077
Indian Journal of Pediatrics	–0.14	39.6%	(=, +, –)	1845	5415
Journal of the American Academy of Child and Adolescent Psychiatry	–0.14	49.0%	(+, =, –)	988	6876
Journal of Pediatrics	–0.15	49.0%	(+, +, –)	4322	26,676
Pediatric Nephrology	–0.15	46.8%	(+, +, –)	1904	11,231
Early Human Development	–0.15	54.0%	(+, =, –)	1651	6896
Journal of Perinatal Medicine	–0.15	41.9%	(+, =, –)	1022	5207
Pediatric Radiology	–0.16	40.2%	(+, +, –)	2323	9539
Journal of Child and Adolescent Psychopharmacology	–0.16	47.2%	(=, +, –)	820	4643
Journal of Perinatology	–0.17	45.7%	(=, =, =)	1965	1338
Pediatric Clinics of North America	–0.17	49.0%	(+, =, –)	821	1704
Pediatric Hematology and Oncology	–0.17	47.9%	(=, +, –)	709	3514
Journal of Clinical Pediatric Dentistry	–0.17	46.7%	(=, =, –)	644	1125
Pediatrics	–0.18	50.8%	(+, +, –)	7111	42,269
Pediatric Infectious Disease Journal	–0.18	48.4%	(+, +, –)	3358	21,214
Journal of Child Neurology	–0.18	50.4%	(+, +, –)	2271	10,296
Journal of Pediatric Endocrinology & Metabolism	–0.18	52.8%	(+, +, –)	1901	8693
Turkish Journal of Pediatrics	–0.18	52.8%	(+, +, –)	1237	5906
Hormone Research in Pediatrics	–0.18	52.7%	(+, =, –)	901	4802
Child Psychiatry & Human Development	–0.18	58.6%	(+, +, –)	709	2909
Fetal and Pediatric Pathology	–0.18	50.4%	(+, =, –)	506	2164
Clinical Pediatrics	–0.19	55.5%	(+, +, –)	1729	6878
Pediatric Pulmonology	–0.2	45.3%	(+, +, –)	1882	9653
European Journal of Pediatric Neurology	–0.20	52.9%	(+, +, –)	1007	5455
Iranian Journal of Pediatrics	–0.20	40.0%	(=, +, –)	893	3332
Ajp Reports	–0.20	47.1%	(+, =, –)	215	986
Archives of Pediatrics & Adolescent Medicine	–0.21	53.1%	(+, =, –)	638	3231
American Journal of Perinatology	–0.22	46.9%	(+, +, –)	1579	7859
Academic Pediatrics	–*0.22*	61.4%	(+, +, –)	864	4267
Journal of Pediatric Gastroenterology and Nutrition	–0.24	45.3%	(+, +, –)	2971	16,886
Neonatology	–0.24	43.2%	(+, +, –)	955	4868
Journal of Pediatric Ophthalmology & Strabismus	–0.24	40.2%	(+, =, –)	557	1932
Children-Basel	–0.24	59.4%	(=, =, –)	216	887
Seminars in Fetal & Neonatal Medicine	–0.25	43.8%	(=, =, –)	428	835
Pediatric Anesthesia	–0.26	39.3%	(+, +, –)	1394	6292
Pediatrics International	–0.28	31.3%	(+, +, –)	2356	12,664
Journal of Aapos	–0.28	43.5%	(=, +, –)	1521	5776
Pediatric Cardiology	–0.30	34.1%	(+, +, –)	2329	10,422
International Journal of Pediatric Otorhinolaryngology	–0.31	39.5%	(+, +, –)	3813	14,151
European Journal of Pediatric Surgery	–0.31	35.3%	(+, =, –)	838	2372
European Journal of Pediatrics	–0.33	48.4%	(+, +, –)	2161	11,267
Pediatric Exercise Science	–0.33	38.3%	(=, +, –)	536	2300
Pediatrics and International Child Health	–0.33	45.3%	(+, =, –)	310	1025
Archives of Disease in Childhood-Fetal and Neonatal Edition	–0.35	46.9%	(=, +, –)	871	3454
Frontiers in Pediatrics	–0.36	47.6%	(=, +, –)	599	3149
World Journal of Pediatrics	–0.36	38.5%	(=, +, –)	587	1949
Clinics in Perinatology	–0.36	43.3%	(=, =, –)	557	1163
Congenital Anomalies	–0.39	31.6%	(=, =, –)	260	1357
Journal of Pediatric Surgery	–0.4	34.3%	(+, +, –)	4960	24,729
Pediatric Neurosurgery	–0.41	23.4%	(=, +, –)	612	2411
Seminars in Pediatric Surgery	–0.42	31.4%	(=, +, –)	500	1176
Journal of Pediatric Orthopedics-Part B	–0.43	19.4%	(–, +, –)	997	3700
Childs Nervous System	–0.49	29.0%	(–, +, –)	2488	10,485
Journal of Pediatric Urology	–0.51	30.7%	(=, +, –)	1557	5407
Archivos Argentinos de Pediatria	–0.51	59.9%	(=, +, –)	392	2042
Journal of Pediatric Orthopedics	–0.57	25.3%	(–, +, –)	1889	8288
Journal of Neurosurgery—Pediatrics	–0.64	24.5%	(=, +, –)	2038	10,457
Italian Journal of Pediatrics	–0.77	53.4%	(=, +, –)	655	3727

The journals are arranged in descending order to their Prestige Index.

The lowest representation of female authors in prestigious authorships are found in the *Italian Journal of Pediatrics* (PI = −0.77), *Journal of Neurosurgery—Pediatrics* (PI = −0.64), and *Journal of Pediatric Orthopedics* (PI = −0.57). In contrast, the best female odds for prestigious authorships are found in the *Journal of Pediatric Health Care* (PI = 0.54), *Journal of Pediatric Nursing—Nursing Care of Children & Families* (PI = 0.57), and *Journal of Perinatal & Neonatal Nursing* (PI = 0.64).

Regarding FAORs, the journals are characterized by almost uniform authorship distributions. In 94 out of 113 journals, we find higher or equal female odds for first- and co-authorships and lower odds for women to hold last-authorships. Three journals (*Child And Adolescent Mental Health, Developmental Neurorehabilitation* and *Journal Of Perinatology*) stand out with a gender-neutral authorship distribution (=, =, =). Three other journals (*Childs Nervous System, Journal of Pediatric Orthopedic*, and *Journal of Pediatric Orthopedic-Part B*) show the most unfavorable FAOR triplet (–, +, –). These journals are also characterized by low Prestige Indices (PI = −0.49, −0.57, −0.43) and relatively low FAPs (FAP = 29%, 25.3%, 19,4%).

Indeed, the journal’s FAP and Prestige Index correlate strongly (*r*(101) = 0.74, *P* < 0.01) (Supp. Fig. 3). Interestingly, no linear correlation is found between a journal’s 5-Year-Impact-Factor and (a) FAP (r(101) = 0.1, *P* > 0.05) or (b) Prestige Index (*r*(101) = 0.1, *P* > 0.05).

### Differences among subject areas

On the level of subject areas, the FAP values yield between 23.3% in *Orthopedics*, 30.5% in *Surgery*, and 34.8% *Cardiovascular System & Cardiology* and 69.7% in *Rehabilitation*, 78.8% in *Nursing*, and 83.5% in *Health Care Sciences & Services* (Table 3).

**Table 3:** Classification by journals’ subject areas.

Subject area	Prestige Index	Proportion of female authorships	FAOR triplet	No. of articles	No. of authorships
Nursing	0.33	78.8%	(+, –, –)	3520	11,656
Public, Environmental & Occupational Health	0.14	60.5%	(+, –, –)	3057	14,060
Health Care Sciences & Services	0.12	83.5%	(+, =, –)	539	1709
Allergy	0.07	48.4%	(+, =, –)	1299	7047
Tropical Medicine	0.06	43.5%	(+, =, –)	1005	3348
Psychology	0.05	57.6%	(+, =, –)	7334	30,665
Rehabilitation	0.04	69.7%	(+, =, –)	1338	4970
Transplantation	0.03	39.1%	(+, =, –)	1715	9502
Obstetrics & Gynecology	0.02	55.5%	(+, =, –)	10,779	37,190
Rheumatology	0.01	55.3%	(+, =, –)	450	2626
Dentistry, Oral Surgery & Medicine	0.00	51.6%	(+, =, –)	2602	6430
Psychiatry	−0.02	52.5%	(+, =, –)	4180	22,095
Dermatology	−0.02	55.4%	(+, =, –)	2294	9044
Pathology	−0.04	50.1%	(+, =, –)	1226	5104
Hematology	−0.06	47.3%	(+, +, –)	7298	42,329
Oncology	−0.07	47.3%	(+, +, –)	7538	42,921
Nutrition & Dietetics	−0.07	48.9%	(+, =, –)	4001	20,944
Behavioral Sciences	−0.07	63.7%	(+, =, –)	838	3807
Immunology	−0.12	48.3%	(+, +, –)	4722	28,293
Endocrinology & Metabolism	−0.12	52.8%	(+, +, –)	4526	22,292
Pediatrics	−0.13	46.6%	(+, +, –)	156,642	690,436
Emergency Medicine	−0.14	43.7%	(+, +, –)	2106	8121
Infectious Diseases	−0.16	48.6%	(+, +, –)	3878	23,750
General & Internal Medicine	−0.16	41.0%	(+, =, –)	2065	10,834
Radiology, Nuclear Medicine & Medical Imaging	−0.17	40.1%	(+, +, –)	2527	10,049
Pharmacology & Pharmacy	−0.18	46.6%	(=, +, –)	1130	5571
Respiratory System	−0.22	46.1%	(+, +, –)	2534	11,456
Neurosciences & Neurology	−0.23	41.5%	(+, +, –)	13,208	59,319
Cardiovascular System & Cardiology	−0.23	34.8%	(+, +, –)	4635	19,496
Gastroenterology & Hepatology	−0.24	45.3%	(+, +, –)	3016	16,968
Anesthesiology	−0.24	39.7%	(+, +, –)	1507	6484
Ophthalmology	−0.29	42.5%	(+, +, –)	2133	7802
Otorhinolaryngology	−0.32	39.3%	(+, +, –)	3884	14,243
Urology & Nephrology	−0.33	41.4%	(+, +, –)	3607	16,945
Physiology	−0.33	38.3%	(=, +, –)	536	2300
Sport Sciences	−0.34	37.9%	(=, +, –)	615	2426
Surgery	−0.39	30.4%	(+, +, –)	13,740	60,582
Orthopedics	−0.54	23.3%	(–, +, –)	3373	12,859

The subject areas are arranged in descending order to their Prestige Index.

Lowest odds ratios for women to hold prestigious authorships are found in the subject areas *Orthopedics* (PI = −0.54), *Surgery* (PI = −0.39), and *Sport Sciences* (PI = −0.34). In contrast, the highest Prestige Indices are found in *Health Care Sciences & Services* (PI = 0.12), *Public, Environmental & Occupational Health* (PI = 0.14), and *Nursing* (PI = 0.33).

A gender-neutral distribution of prestigious authorships (PI = 0) is found at the subject area *Dentistry, Oral Surgery & Medicine*, that interestingly also has an almost balanced FAP of 51.6%.

FAOR patterns are highly uniform at the level of subject areas (+, +/=, –) with significantly higher female odds to secure first-authorships in almost all subject areas and higher or equal FAORs regarding co-authorships. Men have higher odds to hold last-authorships in all 38 subject areas. *Orthopedics* displays the most unfavorable FAOR triplet (–, +, –), has the lowest FAP of 23.3% and Prestige Index of −0.54 of this sub-analysis.

**Fig. 2:**
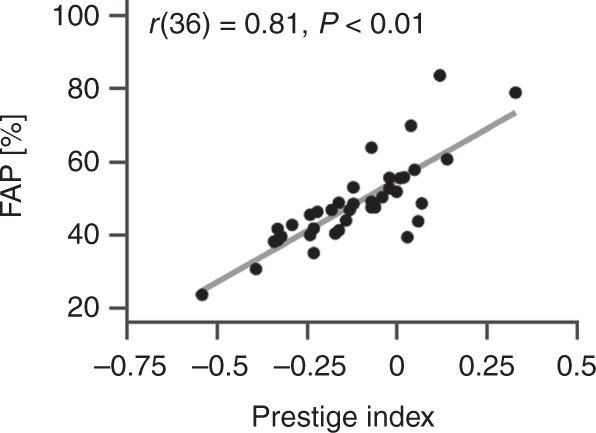
Correlation of parameters in subject areas. The Prestige Index and the proportion of female authorships (FAP) are strongly correlated.

A strong correlation between the FAP and the Prestige Index of a subject area is revealed (*r*(36) = 0.81, *P* < 0.01, Fig. 2).

### Female authorships by the number of authors per article

**Fig. 3:**
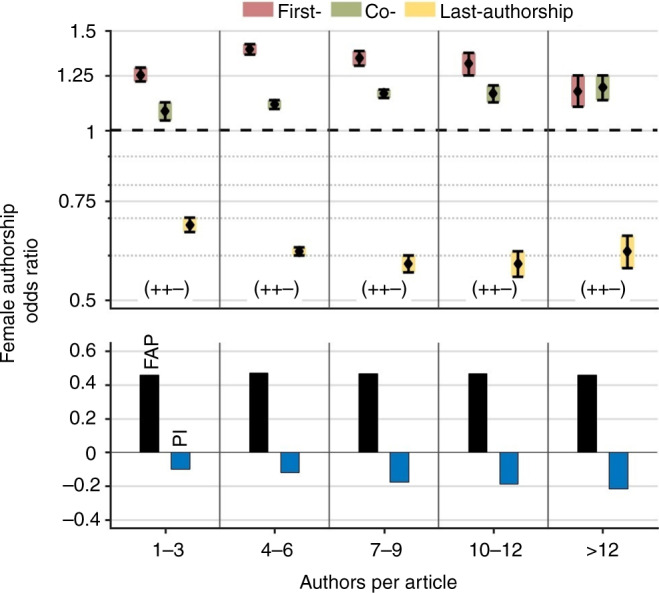
Female authorships by authors per article. With an increasing number of authors per article, the proportion of female authorships (FAP) remains almost constant. In contrast, the Prestige Index (PI) decreases in multi-authored articles due to a female disadvantaged shift of prestigious authorships.

The number of authors per article has little impact on the proportion of female authors. Indeed, the FAP remains essentially stable between 45.7% for articles with 1–3 authors and 47.0% for articles with >12 authors (Fig. 3). However, we find a tendency of increasing female odds for co-authorships and overall slightly decreasing odds for women to hold last-authorships as the number of authors increases. As a result of this subtle drift, the Prestige Index decreases from –0.1 for articles with 1–3 authors to –0.22 for articles with >12 authors. The decline of the Prestige Index displays a female underrepresentation regarding prestigious authorships in multi-author articles. The FAOR triplet remains constant (+, +, –).

### Citation and productivity analysis

**Fig. 4:**
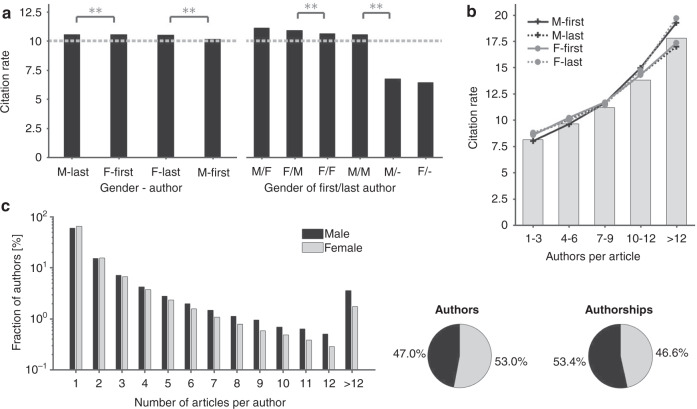
Gender-specificity of citations and scholarly productivity. **a** (left) The descending ordered citation rates reveal only marginal differences between the two genders. The citation rates range from 10.1 citations/article (male first-author) to 10.6 citations/article (male last-author). The dotted line marks the average citation rate of 10.0 citations/article. **a** (right) The analysis of combined authorships reveals that interestingly, mixed key authorships reach significantly higher citation rates than articles with unisex key authorships. Articles published by only one author attract lowest citation rates. **b** The average citation rates by authors per article are depicted ungrouped (bar) and grouped by gender of the key authorships (lines). Citation rates increase with the number of contributing authors. Gender-specific differences in citation rates are minor. **c** (left) Articles per author by gender. Female authors are over-represented in the groups of authors with only one or two published articles, while male authors dominate all other subgroups. **c** (right) The higher productivity of male authors is shown by the fact that 53.4% of all authorships are held by 47% male authors.

Only minor differences are found between the citation rates of female and male authors (Fig. 4a). The average citation rate of all articles in this study (including articles of authors with undetected gender) is 10.0 citations/article. Articles with male first-authorships reach highest citation rates of 10.6 citations/article followed by articles with female first-authorships with 10.5 citations/article. The number of authors is crucial for citation rates. Articles with 1–3 authors, for instance, hold an average citation rate of 8.1 citations per article, while articles with >12 authors achieve an average citation rate of 17.8 citations/article (Fig. 4b).

In terms of scientific productivity, the study shows that male authors are more productive than female authors. 47% of the authors in this study’s data set are male and hold 53.4% of the authorships, whereas 53% female authors hold 46.6% authorships (Fig. 4c). The least productive groups of authors publishing one and two articles are dominated by women. The study overall reveals that 64.7% of all female authors publish merely one article over the course of their medical career. In contrast, for all higher productivity levels we reveal an overrepresentation of male authors. The group of most productive authors with >12 published articles comprises 1.7% of all female authors and 3.5% of all male authors (Fig. 4c).

## Discussion

### High participation of women

This descriptive study examines the integration of female scientists by means of scientific authorships in the academic field of pediatrics from 2008 to 2018. In contrast to other medical sub-disciplines,^9–11,13^ this analysis reveals that, in fact, the majority of authors in pediatric research are female (53.0%). Owing to a higher productivity of male authors, women are still slightly under-represented with a global proportion of female authorships of 47.9%. When set in relation to bibliometric data of the whole field of academic science with a FAP of <30%^17^ or other recently evaluated medical fields like research about lung-cancer (31.3%),^9^ prostate cancer (31.7%),^10^ epilepsy (39.4%),^13^ or dermatology (43.0%),^11^ pediatrics stands out with an exceptionally high participation of women. The continuously rising FAP reflects the increasing proportion of women in medicine, particularly in pediatrics.^26^

### Gender-neutrality is partially achieved

Increasing Prestige Indices, climaxing in 2018 with a Prestige Index of –0.05, suggest an approximation to gender-neutrality regarding the distribution of prestigious authorships. Apparently, the results of the citation analysis also point to gender parity. Not only are articles with women in key authorships cited as often as articles with men in key authorships, but the proportion of female authorships also remains high in multi-author articles, which reach the highest citation rates. In this aspect, pediatric research differs strongly from other scientific fields, in which female authors achieve significantly less citations.^12,17^ This finding speaks against an *old boy* (citation-) *network* in pediatric research.

### Female authors yet under-represented in leading positions

Significantly lower female-to-male odds for last-authorships display a lack of women in senior positions in pediatric research. While many young women enter the academic field of pediatrics,^27^ they often leave the scientific career path earlier than men do.^1,7^ This phenomenon is known as the *leaking pipeline.*^28^ For example, in the US, the most productive country in pediatric research (Supp. Fig. 1), women are over-represented at early-career stages, with 71% female residents in pediatrics in 2018.^1^ However, the proportion reduces over the next career steps and only few reach senior leadership positions, reflected by a female proportion of only 27.5% of the department chairs in pediatrics in 2018.^1^ Career dichotomies like this can be found in most academic disciplines and have been examined in many studies.^7,17,28–33^ As research has shown, one major reason for the imbalance is that female graduate students are relatively less likely than men to aspire leadership positions due to differing life priorities, such as parenthood,^28^ caring for the family,^30^ or a satisfying life-work-balance,^34^ but also due to a lack of role models.^35^

Nevertheless, our study reveals that growth rates for female last-authorships are higher than for other authorship types. Fishman et al.^5^, in contrast, detected higher growth rates for female first-authorships than for last-authorships in their study of three pediatric high-impact journals. This difference raises the question of whether the distribution of authorships is affected by the journal’s influence.

However, significantly increasing last-authorship FAORs and high growth rates for FAPs of last-authorships indicate that female scientists, yet under-represented, are on the rise to occupy senior positions in pediatric research.

### Lower female productivity due to differing lifestyle priorities

Overall, the productivity of a scientist is crucial when it comes to funding, tenure, or promotion. Here, large publication records offer an advantage.^30,36,37^

As van den Besselaar et al. have shown for various scientific disciplines, there are typically no significant productivity differences between male and female authors at early-career stages.^30^ A gender gap with higher male publication counts usually appears in the mid-career phase.^30^ However, at latter career stages, female publication numbers rise and can even exceed those of men.^36^

There are multiple reasons for productivity imbalances. One reason can be found in the female underrepresentation in leading positions. Since higher academic rank is associated with high levels of supervision and publication of scientific work and participation in (citation-) networks,^11,36^ female underrepresentation leads to fewer authorships.^38^ Another reason for productivity differences might be found in the fact that young female scientists are often absent from work for at least a small period of time due to child bearing.^30^ In addition, female pediatricians have more household responsibilities than their male colleges^39^ and more than one-third of female pediatricians in the US work part-time.^40^ Interestingly, the gender-related difference in part-time work accentuates at ages 40–49, with 40% of the female and only 5% of the male pediatricians working part-time.^40^ This period matches the less productive mid-career phase. In summary, the underrepresentation in leading positions and differing female lifestyle priorities are two major reasons for lower female productivity.

### Socio-cultural factors cause region-specific differences

We revealed large region-specific differences of gender disparities in pediatric research. The findings are consistent with those of other medical disciplines.^9–12,25^ The Netherlands and the Scandinavian countries Sweden, Norway and Denmark lead the PI rankings in several medical disciplines,^9,11,12^ indicating that they provide the best career opportunities for female researchers.^5^ The opposite applies to countries such as Japan, Italy, and Greece, most of which are at the bottom of the PI rankings.^9–12,25^

Since these findings also correlate with the Global Gender Gap Report (GGGR),^41^ it can be assumed that regional differences are not founded in characteristics of pediatric research, but are rather due to socio-cultural characteristics of the respective countries.^13^ Japan, for example, is in position 110 of all 149 countries in the GGGR 2018 and in position 40 of 42 of our study. The extremely low FAP of only 21.8% and a Prestige Index of –0.77 in Japan can most likely be seen as an expression of the country’s patriarchal and male-dominated structures.^31^

Interestingly, no correlation of a country’s FAP and Prestige Index can be determined (*r* = 0.18, *P* > 0.05), suggesting that a country with a high proportion of female authors might not necessarily offer good career opportunities for female scientists. In Italy, for instance, female authors predominate with a FAP of 55.2%, but the country provides the worst female prospects in our study with a Prestige Index of −0.9. Accordingly, the theory of critical mass, postulating that the structures of a group change in favor of a minority as soon as it exceeds a critical mass,^42^ does not apply on the country-level due to the strong influence of socio-cultural factors.

### Homogeneous structures in pediatric sub-disciplines

The analysis reveals that, unsurprisingly, some pediatric sub-disciplines are clearly male-dominated (e.g., *Orthopedics* FAP = 23.3%), while others are female-dominated (e.g., *Nursing* FAP = 78.8%). These findings agree with the gender distribution of the respective subjects in adult medicine.^1,43^ Fischer et al.^19^ found an underrepresentation of women in Pediatric Orthopedics, too.^19^ However, they detected an increasing proportion of female first-authors from 13.5% in 2005 to 25.6% in 2015, indicating that women are rising in this male-dominated sub-discipline.^19^

Regardless of the large FAP range of pediatric sub-disciplines (∆FAP = 60.2%), there is a high homogeneity in terms of publication opportunities. FAOR patterns show higher female odds to hold first-authorships and lower female odds to hold last-authorships in 34 of 38 subject areas compared to male odds. The high level of uniformity is also reflected by a relatively small PI rage (∆PI = 0.87). The findings suggest that research group structures in almost all pediatric sub-disciplines are characterized by mainly female early-career researchers and mainly male leaders.

The strong correlation between the FAP and Prestige Index of subject areas (*r* = 0.81, *P* < 0.01) implies that with an increasing proportion of female authors, the female odds to hold prestigious authorships rise in the respective subject area. In this case, the finding is consistent with the theory of critical mass.^42^

### Female integration at the journal-level

Journals differ strongly in terms of the proportion of female authors. With a FAP range of ∆FAP = 65.4 the variation of journals is even higher than of subject areas. Nevertheless, again, we find a high degree of homogeneity regarding publication opportunities with mainly higher female odds ratios for first-authorships and higher male odds ratios for last-authorships. The parallels between pediatric sub-disciplines and journals can be explained by the assignment of subjects to partially subject-specific journals. Interestingly, on the journal-level, the PI values diverge more strongly (∆PI = 1.41) with deviations both upwards and downwards than on the subject-level. We suggest that socio-cultural factors lead to the stronger deviation, as some of the examined journals are country-specific. The lowest Prestige Index in the journal-specific analysis, for example, is found in the *Italian Journal of Pediatrics* with a PI of –0.77, which is consistent with the country-specific analysis pointing out Italy as the country with the lowest Prestige Index.

The discovered correlation between the FAP and Prestige Index on the journal-level (*r* = 0.74, *P* < 0.01) reveals the influence that the female share has on the distribution of prestigious authorships in journals.

The 5-Year-Impact-Factor of a journal, however, does not correlate linearly with the FAP (*r* = 0.1 *P* > 0.05) nor the Prestige Index (*r* = 0.1 *P* > 0.05), indicating that the impact of a journal does not affect the integration of female scientists in pediatric research.

### Outlook

In contrast to other fields,^7,12^ the temporal development of pediatric research displays an explicit progression of increasing female odds to secure first- and last-authorships combined with concurrent decreasing female odds for co-authorships. A linear projection of the obtained data forecasts a rising FAP and increasing FAORs for first- and last-authorships in combination with female odds for co-authorships dropping below one (Supp. Fig. 2). This projection results in a switch of the FAOR triplet from (+, +, –) to (+, =, –) and predicts a FAP of 54.0% and a positive Prestige Index of 0.05 in 2023. Thus, further improvement in career opportunities for women in pediatric research can be expected. However, leading positions will still be predominantly occupied by men in the coming years.

### Methodical limitations

The applied method offers the possibility to algorithmically analyze high amounts of data independent of the examiner. As it is frequently used, values like gender-specific odds ratios or Prestige Indices can easily be compared to other medical disciplines.

For articles published before 2007, the method is not feasible, since the author names were predominantly abbreviated with initial letters, making first-name-based gender determination impracticable.^25^ Shared first- or last-authorships cannot be detected by Gendermetrics.Net and were therefore not taken into account.^11^ As already mentioned by other studies,^12,17,25^ variables, such as the academic rank, employment status and age of the author, were not examined due to lack of information. Moreover, it should be noted that also the profession of the author is not considered. Since journals assigned to pediatrics build the data basis, articles of pediatrics faculty published in non-pediatric journals are not included in the analysis. Furthermore, a change of the last name owing to marriage could not be taken into account in the articles-per-author sub-analysis. In addition, China and South Korea were excluded from the country-specific analysis because of the large proportion of unisex names.

The limitations that result from the software-supported analysis can be addressed in further research by individual investigations, particularly on author attributes. Besides, a disclosure of the authors gender in the submitting process could support investigations on gender disparities.

## Conclusion

In the present study, it was shown that the integration of female scientists is advanced in pediatric research, compared to other scientific disciplines.^12,17^ With nearly balanced publication counts between female and male authors in 2018, similar citation rates, and a Prestige Index which is approaching an almost equal distribution of prestige-associated authorships, the gender gap has narrowed over time. Nevertheless, for pediatric research, as for most scientific fields,^7,12^ a gender-based career-dichotomy could be observed, with relatively more female first-authors at early-career stages and mainly male last-authors in leadership positions. According to linear projections, improving career opportunities for women in pediatric research can be expected in the coming years. Further investigations in the future will reveal whether a ceiling effect occurs or whether gender parity is achieved in pediatric research. It is up to working groups and journals to question their structures and discuss if or how they want to contribute to closing the gender gap.

## Supplementary information

Supplementary Information
