# Supplementary material for: Gender disparities in pediatric research: a descriptive bibliometric study on scientific authorships
Source: Pediatr Res. 2022 Mar 28;92(1):80–90. doi: 10.1038/s41390-022-02010-1 (PMC9411057; doi:10.1038/s41390-022-02010-1)
Supplement: Supplementary file 1 — Supplementary Information [file 41390_2022_2010_MOESM1_ESM.pdf]

Gender Disparities in Pediatric Research - Supplementary figures

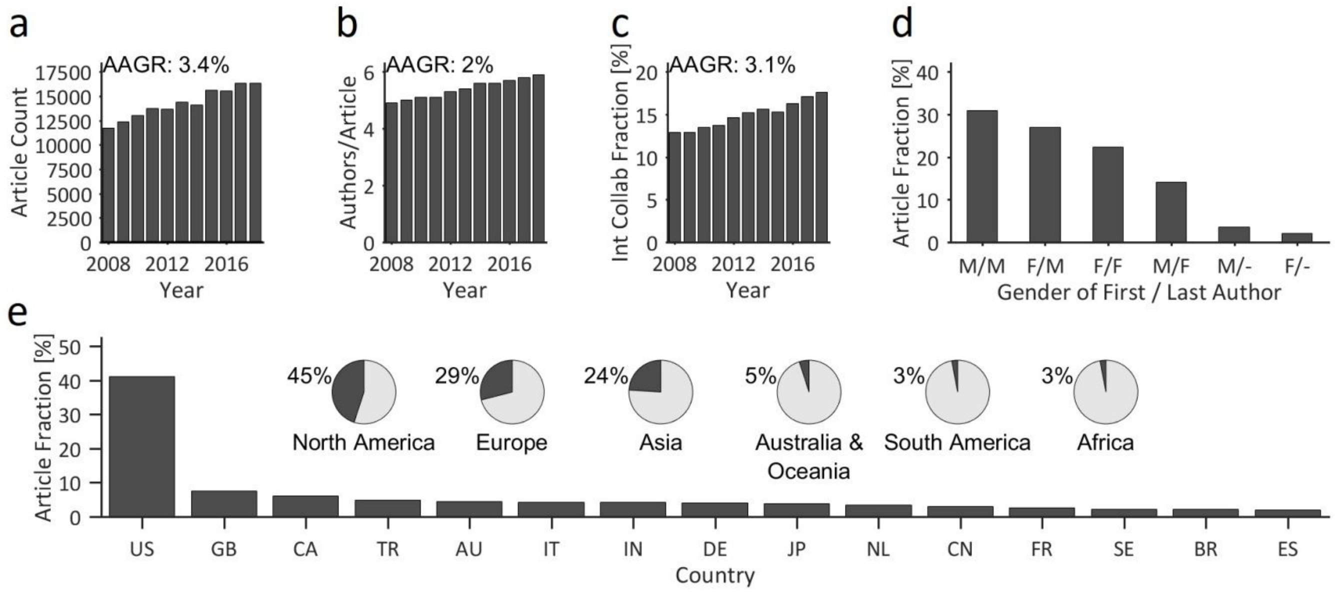

Supp. Figure 1: Overview of published articles worldwide. (a) The number of published articles in the academic field of pediatrics increases from year to year with an average annual growth rate of 3.4%. While in 2008 only 11,741 original articles were published, 16,315 articles were released in 2018. **(b)** The number of authors per article has been ascending from 2008 to 2018. **(c)** A clear tendency of a growing fraction of collaboration articles is exhibited. **(d)** With 30.9% the highest proportion of articles is published by male scientists in key authorships. More male than female researchers publish articles as single authors. **(e)** The fraction of articles is displayed by continents and countries. By far North America, is the leading continent with a share of 45% of all published articles in the field of pediatric research, comprising the US with an overall article fraction of 41%. The second highest productive continent is Europe followed by Asia. Australia & Oceania, South America and Africa stand behind with a fraction of 5% or less.

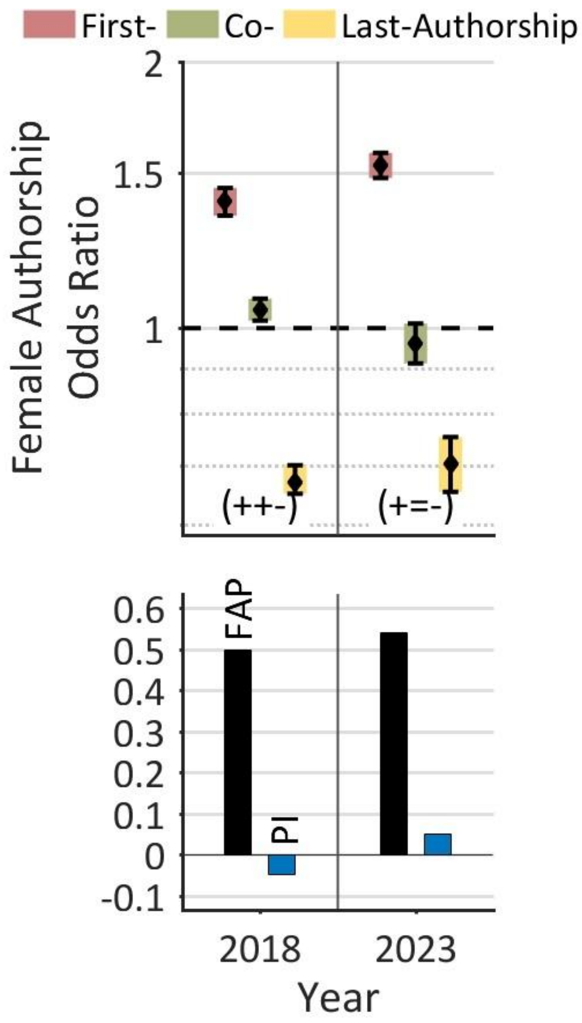

Supp. Figure 2: Linear projection. The prognosis forecasts that the proportion of female authorships (FAP) will continue growing up to 54.0% in 2023. The female odds ratios for first- and last-authorships will increase, while the odds ratios for co-authorships of women will drop below one, resulting in a switch of the FAOR-triplet from (+, +, -) to (+, =, -) and a positive Prestige Index (PI) of 0.05 in 2023.

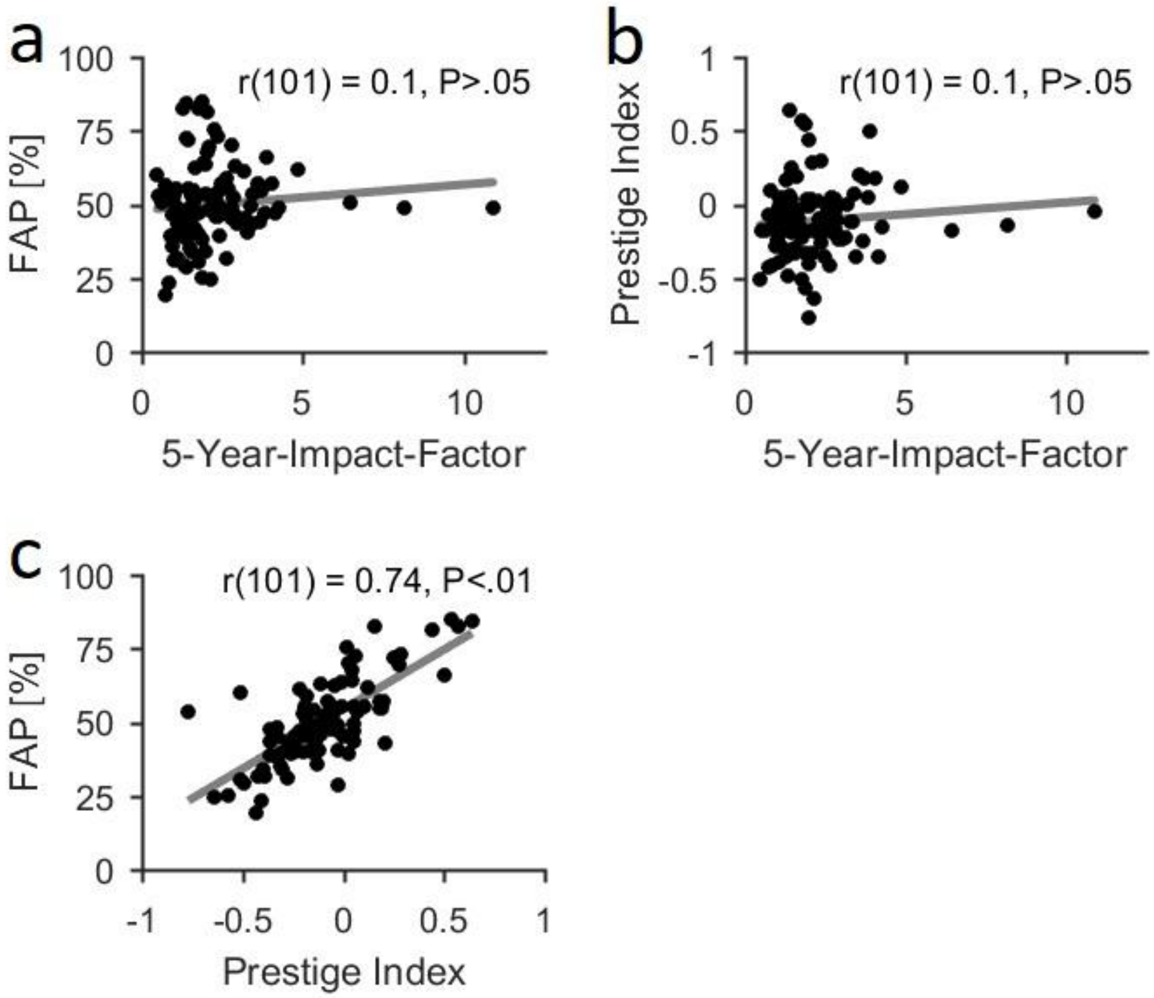

Supp. Figure 3: Correlation of journal parameters. (a, b) There is no linear correlation of a journals 5-Year-Impact-Factor and it's proportion of female authorships (FAP) or Prestige Index found. **(c)** A strong correlation is revealed between the Prestige Index and the FAP of journals.
